# AI-driven sleep apnea screening with overnight blood oxygen saturation: current practices and future directions

**DOI:** 10.3389/fdgth.2025.1510166

**Published:** 2025-04-09

**Authors:** Nhung H. Hoang, Zilu Liang

**Affiliations:** Ubiquitous and Personal Computing Lab, Faculty of Engineering, Kyoto University of Advanced Science (KUAS), Kyoto, Japan

**Keywords:** sleep apnea, SpO_2_, oximeter, mobile health (mHealth), digital health, machine learning, deep learning, apnea-hypopnea index

## Abstract

Sleep apnea is one of the most common sleep disorders, which, if left untreated, may have severe health consequences in the long term. Many sleep apnea patients remain non-diagnosed due to lacking access to medical tests. In recent years, portable and wearable sensors that measure blood oxygen saturation (SpO_2_) are becoming common and affordable for daily use, and they open the door for affordable and accessible sleep apnea screening in the context of everyday life. To learn about the advancement in SpO_2_-based sleep apnea screening, we conducted a survey of published studies. We searched databases including Springer, Science Direct, Web of Science, ACM Digital Library, and IEEE Xplore using the keywords “sleep apnea” AND (“SpO2” OR “blood oxygen saturation”) AND (“machine learning” OR “deep learning”). After screening 835 results, we included 31 publications for a full-text review. Analysis shows that SpO_2_-based sleep apnea screening studies consist of three main categories: (1) individual apnea events detection, (2) apnea-hypopnea index prediction, and (3) apnea severity classification. We found two significant research gaps: a lack of sufficient and diverse publicly available datasets, and the absence of standardized protocols for data collection, signal preprocessing, and model bench marking. Future research should focus on addressing these gaps to enhance the effectiveness and reliability of AI-driven sleep apnea screening methods using SpO_2_ signals.

## 1 Introduction

According to a comprehensive review by Benjafield et al. (1), nearly 1 billion people are affected by sleep apnea, with prevalence exceeding 50% in some countries. Undiagnosed sleep apnea has been shown to be associated with high comorbidities and mortality, and reduced quality of life (2). Many people with sleep apnea do not experience noticeable symptoms, leading to a lack of motivation for diagnostic testing (3, 4). This review assesses recent advances in AI algorithms for the screening of sleep apnea, emphasizing the use of SpO_2_ due to its non-invasive nature and effectiveness, and aims to highlight future research directions.

The rise of home-based sleep apnea tests (HSAT) has highlighted the potential of wearable devices in supporting sleep health in everyday life. Smartwatches such as the Apple Watch, Samsung Watch, Google Pixel Watch, and Fitbit have become popular due to their convenience and functionality (5, 6). These devices generally include a green light reflective photoplethysmography (PPG) sensor for measuring blood oxygen saturation (SpO_2_). Given that sleep apnea impacts both airway and SpO_2_ levels (7–9), many studies have been conducted to explore SpO_2_ as a light alternative to PSG for home-based sleep apnea detection, especially when combined with AI-driven computational methods (10–13). However, there remains a gap in understanding current modelling practices and performance. This mini-review aimed to explore such gaps and potential solutions in sleep apnea detection. Section 2 outlines the review methodology. Section 3 examines employed databases, SpO_2_ processing techniques, feature extraction methods, sleep apnea screening approaches, model development and performance across each screening task. The final section will evaluate remaining limitations and propose future research directions.

## 2 Materials and methods

We followed a search and selection process that is consistent with the methodology for mini-reviews, as outlined (14). Keywords “sleep apnea” AND “SpO2” AND “machine learning” were used to search publications in 4 databases: Science Direct (n=418), ACM Digital Library (n=208), IEEE Xplore (n=25), and Springer Link (n=173), yielding a total of 824 entries. All entries retrieved were imported into Rayyan (15) to streamline the review process and eliminate duplicates. Through Rayyan’s duplication removal feature, 31 duplicate articles were identified and removed. The remaining articles were then screened based on predefined inclusion and exclusion criteria. Inclusion criteria required that articles focus on developing an application, model, or algorithm specifically for the screening of sleep apnea. In addition, studies needed to employ blood oxygen saturation (SpO_2_) signals as a primary input for the screening approach and employ machine learning algorithms as part of the methodology. Exclusion criteria were applied to further refine the selection. Articles were excluded if SpO_2_ was not the main signal used in the algorithm. Studies on pediatric populations were excluded due to the distinct nature of sleep apnea in children compared to adults. Articles that were not publicly accessible or not written in English were also excluded.

Following the title and abstract screening, 52 articles remained. 21 articles were excluded from the analysis: 2 of these excluded articles were identified as review papers, 3 additional articles employed demographic data as the primary input for regression or classification tasks, 9 excluded articles focused solely on apnea event detection using definitions established by the American Academy of Sleep Medicine (AASM), not incorporating machine learning algorithms as required. Finally, 7 articles were excluded due to a lack of relevance to the overall topic. A final review based on the main content resulted in a final selection of 31 articles deemed relevant for this review, the main findings are provided below.

## 3 Results

### 3.1 Sleep datasets

More than half of the studies (n=18) used proprietary datasets collected within research facilities, with dataset size ranging from fewer than 50 sleep records (16, 17) to several hundreds (18–21). While proprietary datasets allow tailored data collection and expert labeling, their limited public availability poses challenges for comparison and benchmarking.

In contrast, public datasets provide valuable alternatives. Widely-used ones include the Apnea-ECG Database (AED) (22), the St.Vincent’s University Hospital/University College Dublin Sleep Apnea Database (UCD) (23), and more recently, the OSASUD dataset (24). However, the relatively small sizes of these datasets (often fewer than 100 records) limit their applicability primarily to epoch-wise, rather than subject-wise screening (8, 9). The Sleep Heart Health Study (SHHS) (25), with over 5,000 recordings and high-resolution labels, is ideal for deep learning models. Other publicly available datasets, such as the Wisconsin Sleep Cohort dataset (n=2,570) (26), Cleveland Family Study (CFS) dataset (n=2,284) (27), Osteoporotic Fractures in Men Study (MROS) dataset (n=3,753) (28) and the Multi-Ethnic Study of Atherosclerosis (MESA) dataset (n=2,002) (29), offer valuable data but have been less utilized in sleep apnea research so far.

### 3.2 Pre-processing SpO_2_ signals

Physiological signals such as SpO_2_ are prone to movement contamination and thus require pre-processing to remove noises. However, our analysis revealed that many studies (n=11) proceed with raw SpO_2_ without pre-processing, and there is a lack of standardized protocols for filtering noise or assessing signal quality.

SpO_2_ are typically calibrated within a range of 70% to 100% saturation, with an accuracy of ±2% to ±4% (30). Consequently, readings below 70% may be inaccurate, prompting some studies to use thresholds of 70% or 65% to remove unreliable readings (3, 31). A lower threshold of 50% has also been used in several studies to account for physiological limitations and equipment errors (9, 12, 17, 20, 32). In addition to cut-off thresholds, some studies applied further noise reduction techniques, such as removing data points where consecutive SpO_2_ values differ by more than a predefined value (e.g., 4%) (3, 12, 20, 33).

Few studies (n=3) explored optimal SpO_2_ signal bands. One study identified the apnea-related band as 0.014–0.033 Hz (33), while (21) argued that the shape of SpO_2_ signal is similar to a sinusoid with 0.02 Hz frequency and therefore used a 0.02 Hz IIR Butter-worth low-pass filter to suppress and smooth the SpO_2_ signal. Another study employed a complex Wavelet filter to eliminate noise from muscle movements (4). Furthermore, Stuban and Niwayama (34) demonstrated that lowering the low-pass filter frequency to a value closer to the fundamental frequency of the PPG signal reduced noise without compromising measurement accuracy. An additional 10 dB of signal-to-noise ratio (SNR) is recommended for accurate SpO_2_ measurement (35). However, no study provided a detailed justification for these methods, nor did they analyzed how these techniques influenced signal quality and subsequent classification performance.

The most common pre-processing practice involves using a threshold between 50% and 100% to eliminate hardware errors. However, to determine the optimal filter settings, future studies are needed to conduct a comprehensive benchmarking of all possible filter options.

### 3.3 Feature construction and selection

Two primary approaches dominate feature extraction methods. The first approach relies on manual feature extraction, leveraging the researchers’ domain expertise. This approach has been applied in 19 studies, where hand-crafted features were derived to ensure model interpretability and applicability across various shallow learning models. While this method can be time-consuming, it remains valuable for its transparency and ease of understanding. A comprehensive list of SpO_2_ features is detailed in Xie and Minn (9), Gutiérrez-Tobal et al. (36), Levy et al. (37).

To optimize features for model construction, several techniques have been employed to select features with strong discriminating power. These include forward stepwise logistic regression (18), recursive feature elimination (38), fast correlation-based filter (36), maximum relevance minimum redundancy (11), and heat-map (39). One commonly used feature is the Oxygen Desaturation Index (ODI), due to its strong correlation with the Apnea-Hypopnea Index (AHI) which is a standard measurement of sleep apnea severity (7, 11, 20, 33, 39–42). However, ODI requires a minimum sampling rate of 1 Hz, which limits its applicability in datasets from lower-frequency devices like smartwatches. Other features such as entropies (7, 12, 40), Lempel-Ziv complexity (LZ) (7, 33, 43), and demographic features (e.g., age, gender, neck circumference, body mass index (BMI)) (41, 44, 45) have also gained prominence. These features have been ranked highly in importance compared to others.

Deep learning (DL) has recently emerged as a novel feature extraction paradigm. DL models, such as convolutional neural networks (CNNs) (46, 47) and long short-term memory (LSTM) networks (8), are capable of automatically extracting features, potentially uncovering patterns that manual feature extraction may overlook. For instance, a study by Lyden et al. (48) demonstrated that CNN and LSTM models achieved high performance in epoch-wise classification, with accuracy, sensitivity, and precision exceeding 90%, even when working with reduced signal sampling rates. This highlights the effectiveness of DL in apnea screening. However, the trade-off is that the interpretability and explainability of these extracted features remain an ongoing challenge as visualizing how the variables are interconnected and weighted within the network is virtually impossible (49).

Manually crafting features is a viable approach to enhancing the explainability and interpretability of machine learning methods, particularly when aiming for clinical acceptance. Unlike features derived from deep learning models, which often suffer from the “black box” problem that limits transparency, handcrafted features are well-defined, easy to visualize, and straightforward to interpret. Feature-ranking techniques, such as SHAP or Grad-CAM, can further facilitate a deeper understanding of how machine learning algorithms work. Ensuring that methods are explainable and transparent not only improves their transition to real-world applications but also enables targeted interventions when the model produces incorrect predictions.

### 3.4 Apnea screening model development

**Figure 1:**
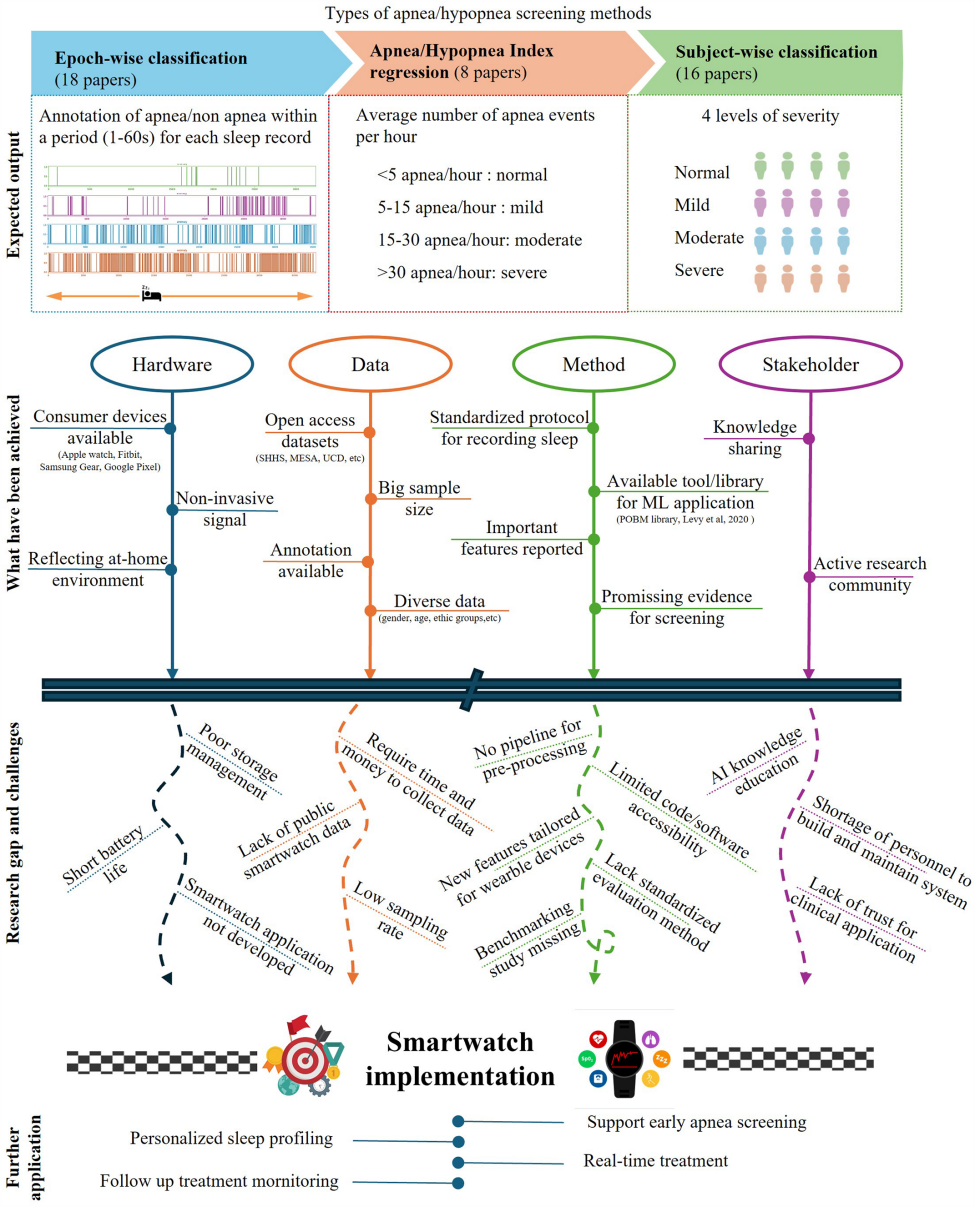
Summary of SpO_2_-based sleep apnea screening in terms of target outputs, primary achievements, research gaps and challenges, and future applications.

All studies in this review employ supervised learning for sleep apnea screening. As shown in Figure 1, the studies were categorized into three main problem formulations, epoch-wise screening, AHI regression, and subject-wise screening.

#### 3.4.1 Epoch-wise model development

Epoch-wise classification, used in 18 studies, involves segmenting a night’s SpO_2_ recording into 1-min epochs, with models classifying each epoch as positive or negative for an apnea event. A few studies framed the problem as multiclass classification to distinguish among normal, hypopnea, obstructive and central apnea events (31, 50). While it is true that sleep apnea screening can be done by calculating AHI by effective respiratory event detection, only 5 studies further post-process the epoch-wise classification results to estimate AHI or assess apnea severity (16, 19, 20, 51, 52).

#### 3.4.2 Subject-wise model development

Subject-wise classification aims to assign a whole night’s SpO_2_ recording either a binary (positive and negative) or a multiclass label (normal, mild, moderate, or severe). This approach classifies the entire night’s recording, eliminating the need to process large number of 1-min epochs, thereby reducing computational cost. While many studies focused on binary classification (7, 12, 36, 38, 43, 53), only a few have applied multi-class classification (40, 54). Another approach is AHI regression, in which a machine learning model aims to predict AHI as a continuous variable, and later categorize sleep apnea severity into predefined classes (e.g., normal, mild, moderate, severe) (11, 33, 38, 52). This approach allows for more granular predictions, which can be particularly useful in identifying borderline cases or tracking changes in AHI over time.

#### 3.4.3 Class balancing

A common issue in sleep apnea classification is the imbalanced distribution among classes. Epoch-wise classifications often have a skewed distribution towards normal epochs [e.g., 90% normal in (4)]. Even with high AHI, normal epochs can dominate [e.g., 69% normal epochs in (16)]. Subject-wise classifications are more balanced but may still skew towards severe cases (e.g., 56% severe in (36)). Several studies address class imbalance with techniques such as random sampling, SMOTE, and ADASYN (10, 21, 33, 38, 44, 45). These methods create synthetic data to balance classes but may alter natural data distribution and impact model generalizability (55).

### 3.5 Model performance in sleep apnea screening

The studies reviewed reveal a combination of classification techniques for the screening of sleep apnea. Table 1 shows various shallow and deep learning models have been applied. However, it is difficult to conclude which method is the most optimal due to the lack of standardized evaluation metrics. For classification problems, evaluation metrics include accuracy (ACC), sensitivity (SE), specificity (SPEC), F1 score, Kappa coefficient, Matthews correlation coefficient (MCC), and area under the curve (AUC). On the other hand, regression problems require a distinct set of evaluation metrics, such as correlation coefficient, intraclass correlation coefficient (ICC), Bland-Altman plots, root mean squared error (RMSE), mean absolute error (MAE), and R-squared. There has yet to be a consensus as to which metrics are the best for evaluating model performance.

**Table 1:** A summary of the dataset used, the signal sampling rate, preprocessing methods, applied models, and result reported in included studies.

Reference	Classification type	Dataset (recordings)	Signal and sampling rate	ML model applied	Pre-processing method	Feature engineering method	Evaluation matrix (result: best)
Alvarez et al. (7)	Apnea/healthy, cut off 10	Proprietary (n=74)	SpO_2_ - 0.2 Hz	KNN^a^, hierachical, fuzzy c-mean		Apprioximate Entropy, Central Tendency Measure, Lempel-Ziv complexity	Acc Sp Se (0.955 0.905 0.833)
Alvarez et al. (18)	Apnea/healthy, cut off 10	Proprietary (n=219)	SpO_2_ - 1 Hz	Logistic regression		Extract and select features using forward stepwise logistic regression	Acc Sp Se (0.870 0.840 0.952)
Xie and Minn (9)	Epoch-wise classification, 1-min-segment	UCD (n=25)	SpO_2_ - 1 Hz ECG - 128 Hz	SVM, KNN, MLP,C4.5 Decision Tree,REPTree,FT Tree,AdaBoost,Decision Stump,Bagging with REPTree^a^ Bagging with Alternative, Decision Tree	Remove criteria: SpO_2_ < 50%	Extract 111 features from ECG and 39 features from SpO_2_	Acc Sp Se (0.844 0.859 0.870)
Zhang et al. (17)	Epoch-wise classification, 5s window	Proprietary (40 records)	SpO_2_ - 1 Hz	SVM	Remove criteria: SpO_2_ < 50%, variation > 10%	Extract 7 features from a window of 150s from the starting point of the SpO_2_ desaturation	Acc Sp Se Effectivity (0.935 0.894 0.957 0.944)
Sánchez-Morillo et al. (40)	4 OSA severity	Proprietary (n=115)	SpO_2_ - 8 Hz	Binary hierarchical classifier		Extract 28 features from SpO_2_	Sp, Se (0.967 0.917)
Hang et al. (20)	ODI detection, AHI regression, 4 OSA severity	Proprietary (n=616)	SpO_2_ - 1 Hz	SVM	Remove criteria: _ SpO_2_ < 50%, _ ΔSpO_2_ > 4%	ODI, neck circumference, BMI, Epworth scalling score	Acc, Sp, Se, AUC (0.901 0.934 0.861 0.952), Bland-altman plot
Mostafa et al. (43)	Apnea/non-apnea, cut off 10	AED, UCD (n=33)	SpO_2_ - 1 Hz	Deep Belief Net		Raw signal	Acc Sp Se (0.976 0.959 0.788)
Jayawardhana and de Chazal (3)	Epoch-wise classification, 1 min segment	Proprietary (n=52)	SpO_2_ - 0.2 Hz	LDA	Exclusion criteria: _ SpO_2_ < 65%, _ ΔSpO_2_ > 4% Moving average filter	32 features from PPG signal 7 features from SpO_2_	Acc Sp Se K (0.85 0.90 0.72 0.61)
Pathinarupothi et al. (8)	Epoch-wise classification, 1 min segment	AED (n=8)	SpO2 - 1 Hz	LSTM - RNN		RNN-based features	Acc Pre Se (0.955 0.992 0.929)
Deviaene et al. (51)	Epoch-wise classification, 1 min segment AHI regression	SHHS, AED, UZ Leuven (n=8,552)	SpO_2_ - 1 Hz	SVM, KNN, LDA, RF^a^	Remove SpO_2_ < 50% linear interpolation Moving average filter Re-annotate label	143 features (Time domain, desaturation severity, statistical, Quasi-periodicity features)	Acc Sp Se PPV AUC K (0.828 0.886 0.643 0.642 0.854 0.527)
Hwang et al. (52)	Epoch-wise classification, 1 min segment AHI regression Subject-wise classification, cut off 5, 10, 15	Proprietary (n=230)	SpO_2_ - 1 Hz	CurveExpert Professional software		raw signal	Acc Sp Se PPV NPV K (0.906 0.872 0.829 0.863 0.886 0.72)
Gutiérrez-Tobal et al. (36)	Subject-wise classification, cut off 5, 10, 15	Proprietary (n=320)	SpO_2_ - 1 Hz	LDA, logistic regression, Bayesian MLP, AdaBoost, AB-LDA^a^		Statistical, spectral, non-linear, and clinical OSA-related features	Acc Sp Se (0.787 0.655 0.889)
Rolón et al. (50)	Normal breathing/ Apnea/Hypopnea	SHHS (n=995)	SpO_2_ - 1 Hz	DAS-KSVD	Linear interpolation Wavelet filters	Discriminant structure dictionaries	Acc Sp Se AUC (0.879 0.883 0.876 0.957)
Ma et al. (32)	Epoch-wise classification, 1 min segment	UCD (n=25)	SpO_2_ - 8 Hz	SVM	Exclusion criteria: _ SpO_2_ < 50%	10 statistic features	Acc Sp Se (0.902 0.941 0.876)
Mostafa et al. (13)	Epoch-wise classification, 1 min segment	AED, UCD, HuGDN2008 (n=103)	SpO_2_ - 50 Hz	CNN		CNN-based features	Acc Sp Se (0.927 0.963 0.874)
Mostafa et al. (47)	Epoch-wise classification, 1 min segment	AED, UCD, HuGDN2008 (n=103)	SpO_2_ - 50 Hz	CNN		CNN-based features	Acc Sp Se (0.942 0.958 0.920)
Rahman and Morshed (38)	Normal/Moderate-severe, cut off 15, AHI regression	SHHS (n=1,000)	SpO_2_, EEG - 125 Hz, ECG - 125 Hz, Sleep stage	logistic regression, random forest, Ada-Boost^a^, SVM, Multi-layer Perceptron	Exclude subjects with central apnea	Percentage of sleep time with SpO_2_ level below 90%, 85%, 80%, 75% HRV features EEG features Feature selection, Min-max scaling	Acc Sp Se (0.934 0.934 0.920), RMSE = 4.6 and R-squared value = 0.71
Li et al. (39)	Apnea/healthy cut-off 5/h	Proprietary (n=181)	SpO_2_ - 1 Hz ECG - 200 Hz	Linear classifier, linear SVM, Complex Tree, RUSBoosted Trees, Logistics Regression, Feed-forward neural network^a^		Mean SpO_2_, Min SpO_2_, ODI	Acc Sp Se AUC (0.978 0.939 0.986 0.97)
Bernardini et al. (16)	Epoch-wise classification, 1s segment, ⇒ AHI evaluation ⇒ 4 OSA severity	OSASUD (n=30)	SpO_2_ - 1 Hz ECG - 80 Hz	CNN-LSTM model	Discard segment with 50% null values	CNN-based features	Acc Sp Se F1 AUC (0.943 0.937 0.951 0.927 0.987)
Piorecky et al. (21)	Epoch-wise classification, 1s segment	Proprietary (n=477)	SpO_2_ - 50 Hz Airflow - 50 Hz	CNN	IIR Butterworth low-pass filter, order 2, cut-off frequency of 0.02 Hz. Shifting SpO_2_ signal by 25s	Apprioximate Entropy, Central Tendency Measure, Lempel-Ziv complexity	Acc Sp Se AUC (0.829 0.842 0.816 0.903)
Gutiérrez-Tobal et al. (33)	AHI regression ⇒ 4 OSA severity	SHHS (n=8762) Proprietary (n=322)	SpO_2_ - 1 Hz	LSBoost (Least Square Boost)		Clinical features, Time domain features, Frequency domain features	ICC 0.924, ⇒ Acc Se Sp PPV NPV (0.919 0.865 0.966 0.956 0.894)
Ganglberger et al. (19)	Epoch-wise classification, 1s segment, ⇒ AHI evaluation ⇒ 4 OSA severity	Proprietary (n=412)	Respiratory signal SpO_2_	Random forest		10 selected features from respiratory signal 1 feature from SpO_2_	Acc Se Pre F1-score ROC-AUC PRC-AUC (0.95 0.85 0.49 0.59 0.83 0.52) ⇒ r-square (0.92) ⇒ Acc 0.8
Sharma et al. (4)	Epoch-wise classification, 1 min segment	AED, UCD (n=33)	SpO_2_ - 100 Hz	RUSBoost Decision Trees^a^ Logistic regression KNN SVM	Butter-worth filters order 6 Wavelet-filter to remove motion artifacts	Wavelet-based Shannon entropy features	Acc Sp Se AUC (0.960 0.958 0.961 0.98)
Albuhayri (46)	Epoch-wise classification, 1 min segment	AED, UCD (n=33)	SpO2 - 100 Hz	CNN		CNN-base features	Acc Sp Se Pre F1-score (0.955 0.957 0.936 0.956 0.946)
Singtothong and Siriborvornratanakul (31)	Epoch-wise classification, 30s segment OSA, CSA, MSA, H-desat, H-arousal	SHHS (n=8,068)	SpO_2_ - 1 Hz PR - 1 Hz	CNN	Exclusion criteria: _ SpO_2_ < 70% Linear interpolation SpO_2_ mean is subtracted	CNN-based features	Acc Se Sp F1-score PPV PR-AUC ROC-AUC (0.822 0.828 0.822 0.478 0.336 0.589 0.904)
Lyden et al. (48)	Epoch-wise classification, 1 min segment	AED (n=8)	SpO_2_ - 100 Hz	random forest, SVM, Logistic regession, KNN, Naive Bayes^a^		CNN and LSTM-based features	Acc Pre Se (0.970 0.972 0.969)
Chen et al. (54)	4 OSA severity	MESA, SHHS, MrOS (n=14,433)	SpO_2_ - 1 Hz	DNN-based model	All sleep records were processed to have the same length (8 h)	DNN-based features	Acc Sp Se Pre (0.805 0.931 0.800 0.818)
Levy et al. (11)	AHI regression 4 OSA severity	SHHS, UHV, CFS, MrOS, MESA (n=12,923)	SpO_2_ - 1 Hz	OxiNet	Exclusion criteria: _ TST < 4 h _ Subjects < 18yrs Delta filter noise removal	CNN-based long,short-range features	ICC F1-score (0.96 0.84)
Bark et al. (10)	Apnea and RERA (respiratory effort related arousals), 30s segment	PhysioNet You snooze you win (n=1,983)	SpO_2_ - 1 Hz, ECG - 200 Hz	1D-CNN-LSTM (SeIANet)	Outlier removal, interpolation, Minmax normalization, Segmentation 30s, overlap 5s	CNN-based features	Acc Sp Se F1-score (0.903 0.892 0.913 0.905)
Liang (12)	Apnea/healthy, Severe/others	SHHS (n=5,786)	SpO_2_ - 1 Hz	Logistic Regression, SVM. Light Gradient Boosting Machine (LGBM)^a^	Exclusion criteria: _ TST < 4 h _ SpO_2_ < 50%, _ ΔSpO_2_ > 4%	Feature construction based on multiscale attention entropy analysis and feature transformation using ICA.	Acc Sp Se PPV NPV F1-score MCC AUC (0.881 0.972 0.460 0.800 0.893 0.579 0.539 0.716)
Bilge et al. (53)	Apnea/healthy Severe/others Severe/mild-moderate	Proprietary (n=115)	SpO_2_ - 40 Hz	ANN (5 layers)		Features extracted at different window lengths	Sp Se PPV NPV AUC (0.90 0.84 0.97 0.76 0.93)

Acc, accuracy; Sp, specitivity; Se, sensitivity; Pre, precision; PPV, positive predictive value; NPV, negative predictive value; AUC, area under the curve; K, Cohen’s Kappa; F1, F1-score; ICC, intraclass correlation coefficient; ROC-AUC, area under the receiver-operating characteristic curve; PR-AUC, precision-recall area under the curve. ^a^:bestMLmodelreported.

#### 3.5.1 Epoch-wise model performance

Epoch-wise classification has shown high performance in sleep apnea events detection. For instance, Lyden et al. (48) reported impressive results using shallow models combined with DL-based features, achieving accuracy of 97.04%, specificity of 97.19%, and sensitivity of 96.94%. This study simulated smartwatch data by adding Gaussian noise to down-sampled SpO_2_ signals at various signal-to-noise ratios. Most models, except SVM, were minimally affected by reduced sampling frequency, although performance dropped significantly below a 30dB signal-to-noise ratio. Naive Bayes models with LSTM-based features provided the most stable performance.

A major challenge in epoch-wise classification is the delay between sleep apnea events and SpO_2_ desaturation, with delays reported from 10 to 40 s (21, 51, 56). In addition, subjects sleep only 75.9% of the time, and hypopnea events are more common than apnea events or periods of normal breathing (e.g., 73% in the SHHS dataset). Notably, 11.5% of apneic events lack SpO_2_ desaturation (51), complicating model accuracy. To address these issues, Bernardini et al. (16) focused on detecting clusters of anomalies, providing valuable clinical insights despite lower performance metrics (81.5% ACC, 67.2% SE). The study accurately classified sleep apnea severity in 21 out of 30 cases. Punjabi (57) highlighted that the distribution of apneic events over the night is crucial for understanding their health impact.

In an effort to reduce classification errors, Bark et al. (10) developed a selective model that improves accuracy by rejecting low-confidence predictions, achieving 90.26% ACC, 91.29% SE, and 89.21% SPEC. Despite these promising results, model generalization remains a concern due to small sample sizes. Analysis suggests that RNN and LSTM models generally outperform CNNs in this field (10, 16, 48, 55).

#### 3.5.2 Subject-wise model performance

The OxiNet model by Levy et al. (11) demonstrated high performance and generalization, analyzing 12,923 PSG recordings from multiple databases. Despite a slight decrease in performance on external datasets, the model achieved an F1-score above 0.75 and an ICC greater than 0.92. Subject-wise classification models generally excel at distinguishing severe sleep apnea but struggle with lower severity cut-offs (12, 33). For instance, Levy et al. (11) reported a high misclassification rate for healthy subjects as mild apnea, particularly in the MrOS dataset (44% misclassified). Similarly, Liang (12) achieved better performance with a 30/h cut-off than 5/h, but still faced issues with misclassification. The model by Gutiérrez-Tobal et al. (33) had high sensitivity but lower specificity, indicating a tendency to overestimate severity.

Performance issues may arise from imbalanced data and binary cut-off thresholds. Ganglberger et al. (19) suggested adjusting the AHI threshold could better reflect severity, particularly near the borderline. Studies adjusting the cut-off to 10/h showed more balanced performance (7, 18, 43).

Few studies address post-processing, which is important for identifying and correcting issues not evident during initial model development. Papini et al. (58) proposed a post-processing step to reassess results based on severity discrepancies, considering factors like cardiac comorbidity and medication.

## 4 Discussion

Our analysis examined key aspects of AI-driven SpO2-based sleep apnea screening, including commonly used datasets, signal preprocessing methods, feature extraction and selection, and model performance. Although the results are promising, the variability in devices, algorithms, and study designs makes it difficult to draw definitive conclusions about which devices and algorithms represent the state-of-the-art. Based on our findings, we discuss the research gaps and opportunities associated with sleep apnea screening at home using SpO2 measurements.

### 4.1 Research gaps

Our analysis identified two major research gaps in AI-empowered sleep apnea screening using SpO2 measurements. The first gap pertains to the limitations in the quantity and diversity of datasets. Specifically, there is a scarcity of large-scale, open-access datasets. Large volumes of training data are essential for developing robust AI models, and sharing open datasets is increasingly crucial for advancing research. However, we found that most studies used proprietary datasets that are not publicly available. Despite our focus on wearable deceives for apnea detection, we identified only four datasets collected with such devices (19, 36, 53, 56), none of which are openly accessible. In addition, most of the open sleep datasets were collected in Western countries and predominantly included data from Caucasian individuals. The lack of data from diverse populations, especially Black, Hispanic, and Asian groups, poses a challenge to developing generalizable AI models across different demographic groups (11, 39).

Furthermore, in the context of home-based or self-tracking, the user range has expanded beyond patients to include healthy, young individuals in everyday life. The innovation of portable and wearable sensors has highlighted the urgent need for datasets derived from smartwatches, smart rings, and similar devices. The closer these datasets reflect real-world conditions, the more effectively machine learning algorithms can be applied in practice. Another promising trend is the use of longitudinal data, which provides a more detailed and stable representation of health status over time. In the near future, sleep apnea data from healthy young individuals, collected through consumer devices over extended periods, will be essential for advancing research and improving screening models.

The second major gap is the need for standardization in data collection, signal preprocessing, and model benchmarking. This lack of standardization creates challenges for reproducibility, comparability and generalizability. Data collected across different studies vary significantly due to differences in the type of devices used, the protocols followed (e.g., timing, duration, sensor placement), and the environmental conditions under which data are gathered. Signal preprocessing is another area where standardization is absent. Steps such as noise filtering and artifact removal are often performed differently across studies, leading to inconsistencies in the data quality. In addition, variations in datasets used for model training and testing, coupled with differences in evaluation metrics, hinder direct comparisons between models (19). Many studies focus solely on accuracy (e.g., (11, 31, 47)), which can be misleading, especially in the presence of unbalanced data. The generalizability of models is frequently overlooked; only one study extensively investigated model performance on different datasets other than the training set (37). In contrast, several studies reported decreased model performance when applied to new datasets (21, 31, 46, 47). To facilitate meaningful cross-model comparisons, sharing source code is recommended; however, only a few studies have done so (21, 37, 45).

### 4.2 Opportunities and future directions

To address the identified research gaps in AI-powered sleep apnea screening using SpO2 measurements, future research should focus on enhancing dataset quality and standardization. Developing and sharing large-scale, open-access datasets that include diverse populations is crucial for training robust and generalizable AI models. Collaborative data initiatives involving academia, industry, and healthcare organizations can facilitate the creation of comprehensive datasets. Additionally, establishing standardized protocols for data collection and signal preprocessing will improve reproducibility and comparability across studies. Formulating and disseminating guidelines for data collection and preprocessing, along with creating benchmarking frameworks, can help ensure consistency and facilitate meaningful cross-study comparisons.

Moreover, advancing model evaluation and generalizability is essential for improving AI performance in sleep apnea screening. Future research should emphasize evaluating models on diverse datasets and adopting robust evaluation metrics that account for data imbalance and biases. Encouraging transparency by sharing methodologies, preprocessing scripts, and source code can enhance reproducibility and foster a more reliable research environment. Exploring multi-modal data approaches and innovative sensor technologies could also improve the accuracy and robustness of models. By addressing these areas, the field can make significant strides toward developing more effective and generalizable AI solutions for sleep apnea detection.

Finally, research on AI-based sleep apnea research has been lacking a user-centered perspective, and this needs to be addressed in future studies. In addition to improving model performance, future research must also prioritize clinical applicability and user adoption (59). Keeping experts involved by collecting their feedback to continuously retrain the models can help better align the inner workings of the models with expert decision-making processes. Engaging with both patients and clinicians through pilot studies, usability testing, and observational trials can provide valuable insights into model interpretability, user trust, and practical deployment. Last but not the least, designing user-friendly interfaces that visualize model predictions in an easy-to-understand manner is important for fostering user acceptance and real-world applicability.
